# Electrical and Thermal Properties of Carbon Nanotube Polymer Composites with Various Aspect Ratios

**DOI:** 10.3390/ma15041356

**Published:** 2022-02-12

**Authors:** Dong-Kwan Lee, Jongchan Yoo, Hyunwoo Kim, Byung-Ho Kang, Sung-Hoon Park

**Affiliations:** Department of Mechanical Engineering, Soongsil University, 369 Sangdo-ro, Dongjak-gu, Seoul 06978, Korea; kd2890@naver.com (D.-K.L.); dbwhdcks39@naver.com (J.Y.); hyunwoi@naver.com (H.K.); royce2080@naver.com (B.-H.K.)

**Keywords:** carbon nanotube, composite, thermal conductivity, electrical conductivity, aspect ratio

## Abstract

In response to the rising need for flexible and lightweight materials capable of efficient heat transport, many studies have been conducted to improve the thermal properties of polymers via nanofillers. Among the various nanofillers, carbon nanotubes (CNTs) are considered as the most promising, owing to their excellent thermal and electrical properties. Accordingly, CNT/polymer composites can be used as flexible and lightweight heat transfer materials, owing to their low density. In this study, we fabricated multi-walled CNT (MWCNT)/polymer composites with different aspect ratios to investigate their effects on electrical and thermal properties. Through a three-roll milling process, CNTs were uniformly dispersed in the polymer matrix to form a conductive network. Enhanced electrical and thermal properties were observed in MWCNT composite with a high aspect ratio as compared to those with a low aspect ratio. The thermal conductivity of composites obtained as a function of the filler content was also compared with the results of a theoretical prediction model.

## 1. Introduction 

Polymers are known as effective insulators, owing to their low thermal and electrical conductivity. Research has been actively conducted to improve their electrical, mechanical, and thermal properties and to develop new functions by adding nanoscale fillers to polymer materials [1,2,3,4]. Among the various nanofillers, carbon-based fillers, such as graphene, carbon black, and carbon nanotubes (CNTs), have drawn significant attention, owing to their outstanding physical properties [5,6,7]. Since their discovery, CNTs have been studied as fillers for polymer composites because of their one-dimensional geometry. The high aspect ratio of CNTs forms a conductive net inside a polymer matrix, which is an insulator, to provide electrical conductivity or improve thermal properties [8]. In addition, CNTs are chemically stable because their carbon atoms form sp^2^ covalent bonds in the form of a honeycomb [9,10]. CNTs are classified as single-walled CNTs (SWCNTs) with only one cylindrical wall or multi-walled CNTs (MWCNTs) with multiple cylindrical walls [11]. Previous studies have shown that MWCNTs and SWCNTs have a thermal conductivity of 3000 W/mK and 2000 W/mK, respectively [12]. Because MWCNTs have better thermal properties than SWCNTs and similar metallic properties, they are more advantageous for improving the thermal properties of a polymer composite.

The performance of electronic devices, such as CPUs and GPUs, that we use on a daily basis is greatly affected by heat [13,14,15]. When electronic equipment like mobile phones and laptops are used, heat is a major cause of not only degradation of electronic equipment performance but also shortening of life; thus, it is critical to quickly release heat from electronic devices. Even though polymers have been extensively used in various electronic fields due to their light weight, flexible shape deformation, and mechanical properties [16,17], most polymer materials have poor thermal conductivity or thermal diffusivity compared to metals, which may adversely affect the performance of electronic devices. Thus, studies for improving the thermal properties of a polymer by adding a nanofiller to fabricate composites have been actively conducted [18,19,20].

The electrical and thermal properties can vary significantly depending on the aspect ratio, which is the geometric variable of CNTs [21]. Polymer composites in which CNTs are dispersed homogeneously have points of contact between individual CNTs, which are gathered to form an electrical path that then functions as a percolation network [22,23]. In this case, the tendency of the percolation network to be built is dependent on the CNT aspect ratio, which can also improve thermal conductivity [24,25,26]. Hong et al. [27] produced various polymer composites, such as PDMS and epoxy with CNT and carbon fiber, which are representative one-dimensional carbon fillers, to explore thermal properties and present their thermal behavior. Guo et al. [28] also reported that the overall thermal conductivity of the polymer composite was improved when silica was coated on the outer wall of the MWCNT in multiple layers. Xiao et al. [29] found that uniform dispersion of CNTs is important for enhancement of electrical and thermal properties when studying the dispersion morphology of CNTs inside CNT polymer complexes and also found that the length efficiency of CNTs reveals the behavior of thermal properties through morphological analysis of the complex. In addition, Caradonna et al. [30] discovered CNTs are more advantageous on conductive networks through discovering the electrical and thermal behavior of filler shapes by adding three types of carbon-based fillers with different shapes to a polymer material using CNTs, graphite, and graphene. In the current study, to determine the behavior of the electrical and thermal properties of CNT polymer composite according to CNT aspect ratio, three types of MWCNTs with different aspect ratios were prepared to fabricate three different polydimethylsiloxane (PDMS) MWCNT composites. Scanning electron microscopy (SEM) images were analyzed to determine the length and distribution of MWCNTs in the polymer composites. Then, the electrical and thermal properties, with different aspect ratios of the MWCNTs in each composite, were measured and analyzed by theoretical models.

## 2. Materials and Methods

### 2.1. Materials and Fabrication

Short MWCNTs (S-MWCNTs, CM95, Hanwa Nanotech, Seoul, Korea), middle-length MWCNTs (M-MWCNTs, CM150), and long MWCNTs (L-MWCNTs, CM250) were used as conducting fillers. PDMS (Sylgard 184, Dow Corning, Midland, MI, USA) was used as the base polymer matrix. PDMS and MWCNTs were mixed in a paste mixer (Daehwa, Seoul, Korea) at 500 rpm for 30 s and 1500 rpm for 60 s. After the premixing process, each MWCNT was evenly dispersed into the PDMS through the three-roll milling process (Intech, Seongnam-si, Gyeonggi-do, Korea). The dispersion process was conducted in each PDMS/MWCNT paste with different lengths for 5 min at 100 rpm. The PDMS/MWCNT composites were fabricated by the hot-pressing method (Qmesys Inc., Uiwang-si, Gyeonggi-do, Korea) at 150 °C for 60 min in 15 MPa. In this way, we fabricated 1 mm thick composite films by length at a mass ratio of 10 wt%, and L-MWCNTs were additionally prepared at 1 wt%, 2 wt%, 5 wt%, and 7 wt%.

### 2.2. Electrical Properties

Before measuring the electrical conductivity, an accuracy enhancement process was conducted. First, UV etching (JSE Co., Seoul, Korea) was applied to the surface of the composite film for 300 s to expose the MWCNTs embedded beneath the surface of the PDMS matrix. Then, to form the electrode, the silver paste covered the four points of the composite film at equal distances. Next, the curing process was conducted in an oven at 100 °C for 1 h, and the four-point method (Keithley 487 picoammeter and Keithley 2400 sourcemeter, Keithley, Cleveland, OH, USA) was used to measure the resistance of the composite films without surface resistance.

### 2.3. Thermal Properties

**Figure 1:**
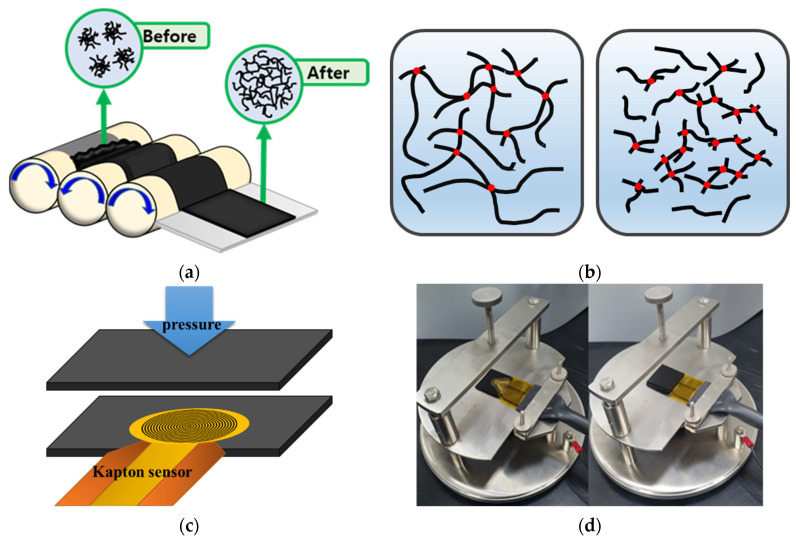
(**a**) Three-roll milling process for dispersion of MWCNTs. (**b**) Scheme of the conductive network of L-MWCNTs and S-MWCNTs. (**c**) Scheme of the measurement of thermal properties with a Kapton sensor. (**d**) Photograph of the measurement of thermal properties.

Thermal conductivity and diffusivity measurements were conducted by the transient plane source (TPS) method with TPS-2500s (HotDisk, Sweden) in compliance with the ISO 22007-2 standard. Each PDMS/MWCNT composite film was cut into 30 × 30 mm^2^ pieces and placed on the test jig. As shown in Figure 1c,d, the Kapton sensor was placed between two pieces of composite of the same size, pressed to reform the surface thermal resistance, and then covered with an insulation cover. Subsequently, 80 mW of power was supplied to the composite pieces through the Kapton sensor for 2 s. The Kapton sensor consists of a double-spiral structure of thin nickel wire coated with protective material against external deteriorating factors. The nickel wire began generating heat as current flowed through it, and the heat was transferred to the material to be measured. As the temperature of the nickel wire changed over time, the resistance also changed, which is expressed by the following equation [31]:

(1) $$R\left(t\right)=R_{0}[1+a\bar{\Delta T\left(\theta \right)}],$$

where *R*_0_ is the initial resistance of the nickel wire pattern embedded in the sensor, $\bar{\Delta T\left(\theta \right)}$ is a geometrical function of the temperature change of the Kapton sensor over time, *a* is the temperature coefficient (TCR), and *θ* is defined as in [31].

(2) $$\theta =\sqrt{\frac{t}{\sigma }},\;\sigma =\frac{r^{2}}{\alpha },$$

where *t* is the heating time, *α* is the thermal diffusivity of the measured sample, and *r* is the radius of the nickel structure. As the resistance of the sensor changed over time, the temperature change was also measured on countless small strips of the sensor. $\bar{\Delta T\left(\theta \right)}\;$in Equation (1) is a function of the integration of the strips where the temperature change was detected. The thermal conductivity and product of the specific heat and density are also involved in the integration function. Thus, the thermal properties of the sample composite can be obtained by integrating the results of each strip [32].

## 3. Results and Discussion

**Figure 2:**
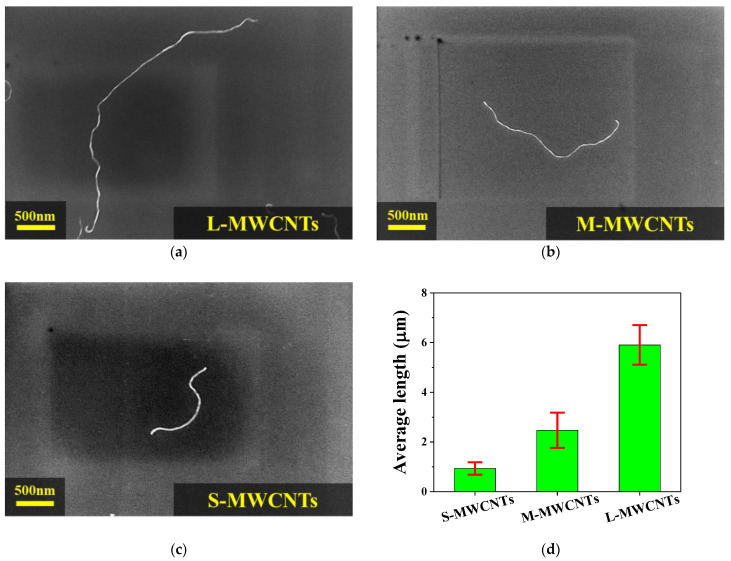
SEM images of three different MWCNT lengths: (**a**) L-MWCNTs, (**b**) M-MWCNTs, (**c**) S-MWCNTs, and (**d**) the average length of each MWCNTs.

CNTs are basically one-dimensional graphene structures rolled into a cylindrical shape. Therefore, their geometric property, i.e., the aspect ratio, has a significant effect on the overall properties of CNT/polymer composites. Figure 2a–c shows SEM (Gemini SEM 300, ZEISS Inc., Land Baden-Württemberg, Germany) images of single MWCNTs with different aspect ratios. To measure the length of the MWCNTs, a small amount of MWCNT powder was dispersed in 100 mL chloroform by sonication for 30 min. The dispersion was then spin-coated onto the silicon wafer at 1500 rpm for 1 min and 20 s, and the length of each MWCNT was measured. In this way, several SEM images were obtained for each MWCNT, and length values of several MWCNTs were calculated; the average value is shown in Figure 2d. Therefore, we calculated the aspect ratio (L/d) of MWCNTs for each length using information on the average length (L) of MWCNTs obtained through experiments and the diameter (d, S-MWCNTs ≈ 12.5 nm, M-MWCNTs ≈ 12.63 nm and L-MWCNTs ≈ 17.06 nm) of the same kind of MWCNTs obtained in previous studies [33,34]. The aspect ratio of S-MWCNTs was found to be 60, whereas it was 173 for M-MWCNTs and 400 for L-MWCNTs.

**Figure 3:**
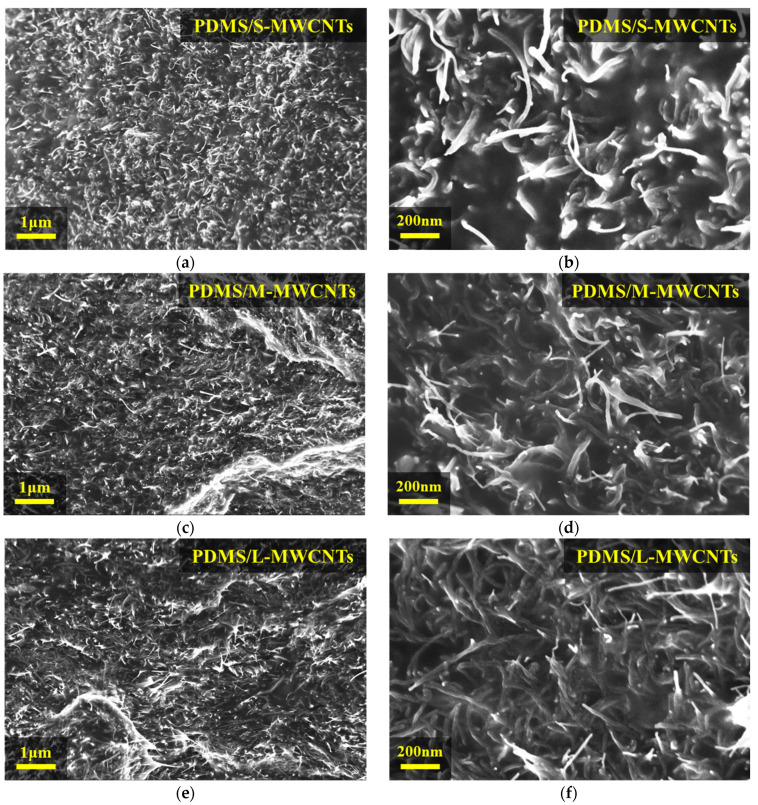
SEM images of PDMS/MWCNTs 10 wt% composites with three different aspect ratios: PDMS/S-MWCNTs 10 wt% composites at (**a**) 10 k and (**b**) 50 k magnification. PDMS/M-MWCNTs 10 wt% composites at (**c**) 10 k and (**d**) 50 k magnification. PDMS/L-MWCNTs 10 wt% composites at (**e**) 10 k and (**f**) 50 k magnification.

In general, the MWCNT length was decreased by sufficient three-roll milling time, resulting in a relatively low aspect ratio of all MWCNTs [23]. Figure 1a shows that the MWCNTs were dispersed in the polymer matrix by the three-roll milling process. In the space between the cylinders of the three rolls, the high shear stress was transferred to the MWCNT bundles entangled by van der Waals forces. These bundles were disentangled for homogeneous dispersion in PDMS. Consequently, we observed the uniform dispersion condition of each composite with different aspect ratios in the SEM images with 10 wt% mass ratio, as shown in Figure 3a–f. We determined that high contents of MWCNT are randomly dispersed in the polymer matrix through the three-roll milling method (see Figure 3a,c,e) at 10 k magnification. Furthermore, the morphological structure of the composite according to the aspect ratio of MWCNTs was also found through SEM images. 

As shown in Figure 3b,d,f at 50 k magnification, it was confirmed that the shorter the length of the MWCNTs, the greater the interval between the MWCNTs. After analyzing the SEM images, we measured the electrical conductivity of 1 wt% and 10 wt% composites for each CNT length. In general, homogeneous dispersion affects the mechanical properties of composites because the aggregation of MWCNTs attenuates the load transfer from the polymer matrix to the MWCNTs [35,36]. From the perspective of electrical conductivity, it can also lead to a better-connected electrical network of MWCNTs in the polymer matrix, owing to the homogeneity of the MWCNT dispersion. Even if the CNTs are not in direct contact inside the polymer matrix, they can exhibit electrical transport, owing to the electron tunneling effect. In a polymer composite in which CNTs are evenly dispersed, electrons can hop from one CNT to another when the distance between CNTs is at the nanometer scale, even though the polymer is an insulating material [37].

**Figure 4:**
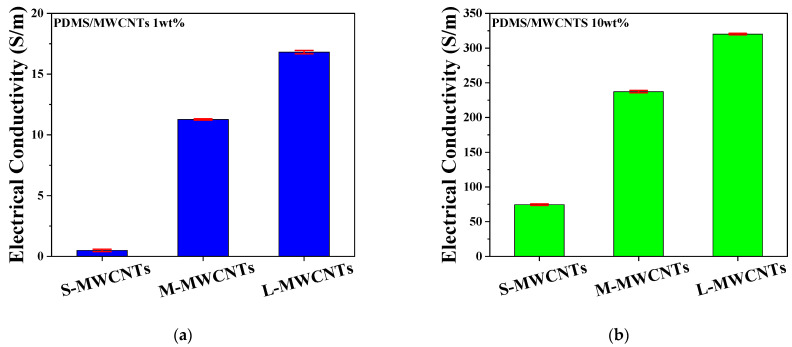
Electrical conductivity data of (**a**) PDMS/MWCNT 1 wt% composites and (**b**) 10 wt% composites with three different aspect ratios.

Electrical conductivity was measured by producing several samples of PDMS/MWCNTs composites of 1 wt% and 10 wt% for each length, and the average values are illustrated in Figure 4. As shown in Figure 4a,b, L-MWCNT composite tends to have better electrical conductivity than S-MWCNT composite. L-MWCNT has the advantage of effective formation of an electrical path, as it is longer than M-MWCNT and S-MWCNT. In the case of S-MWCNT composites, building an electrically conductive network requires a relatively large number of MWCNTs, with sufficient space between each other to induce the electron tunneling effect. Additional contact points are also required compared with L-MWCNT composite polymers, causing large contact resistance. In contrast, L-MWCNTs do not require as many contact points as S-MWCNTs and M-MWCNTs, since their length is sufficient to carry the current by themselves [38]. In Figure 4b, the 10 wt% composites show significantly better electrical conductivity compared with the 1 wt% composites, owing to the greater number of conductive fillers in the polymer.

**Figure 5:**
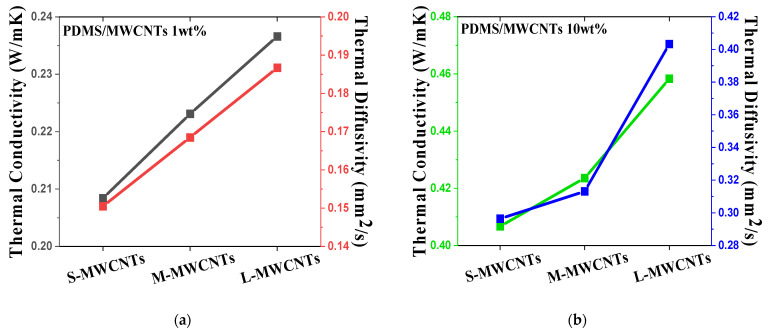
Thermal conductivity and diffusivity data of (**a**) PDMS/MWCNT 1 wt% composites and (**b**) 10 wt% composites with three different aspect ratios.

Moreover, homogeneous dispersion also contributes to the enhancement of thermal properties. The conductive network also plays a key role in enhancing thermal conductivity and diffusivity. The heat conduction is based on the principle that the vibration of the atomic lattice is transmitted to another lattice next to it and vibrates. The polymer matrix is a structure in which chains with high molecular weight are complexly bonded, and in this structure, as the lattice vibration of atoms passes through a disordered molecular structure, the vibration is not completely transferred. On the other hand, since MWCNTs have a regular crystalline structure, vibration is not scattered but quickly transmitted from one end to the other [39]. In CNT polymer composite, thermal resistance occurs at the interface between CNTs and polymers. According to Wang et al. [40], CNTs and polymer have incomplete contact states, which results in phonon scattering at the interface. In the crystal structure of CNTs, as the vibration of the lattice quickly transferred in the longitudinal direction is transferred to the incomplete contact interface with the polymer chain, the polymer molecules vibrate disorderedly, forming phonon scattering. Figure 5a,b shows that L-MWCNTs can significantly improve thermal conductivity and diffusivity compared with S-MWCNTs as polymer fillers in the same context as electrical conductivity. Individual MWCNTs can function as excellent heat transfer mediums because of their superior thermal conductivity (3000 W/mK) [12]. In contrast, S-MWCNTs with the same weight ratio have more individual MWCNTs per unit volume to reach the percolation network, resulting in an increased contact resistance, as shown in Figure 1b. As the number of contacts points increases, the thermal resistance in the interphase region between the MWCNTs and polymer also increases, as scattering of the heat transfer is mediated by phonons [41]. However, MWCNTs cannot make direct physical contact between themselves and are surrounded by the polymer matrix. According to the thermal conduction mechanism as mentioned above, MWCNTs, which have a crystalline structure with excellent thermal conductivity in the longitudinal direction, can almost completely transmit the vibration of the atom lattice. However, short MWCNTs not only have a shorter complete path than L-MWCNTs, but also have more incomplete contact with the polymer. Therefore, when the vibration of the lattice passes through its contact point, phonon scattering occurs, and the speed of heat conduction along the polymer chain is significantly reduced. It is interpreted that short MWCNTs at the same content have more contact resistance.

The observation of the thermal conductivity and diffusivity of the PDMS/MWCNT composites for each aspect ratio showed that L-MWCNTs have the best thermal properties; hence, the thermal conductivities of PDMS/L-MWCNT 1 wt%, 2 wt%, 5 wt%, 7 wt%, and 10 wt% composites were measured to see its trend. Furthermore, we used the thermal conductivity prediction model of CNT polymer presented by Deng et al. [42] and the aspect ratio of L-MWCNTs obtained in previous experiments to confirm how consistent the experimental values and predictions were. The presented predictive model was expressed by modifying the effect of the filler aspect ratio in the composite, and the geometric behavior of MWCNTs was also included by introducing an efficiency length, representing the straightness of MWCNTs.

**Figure 6:**
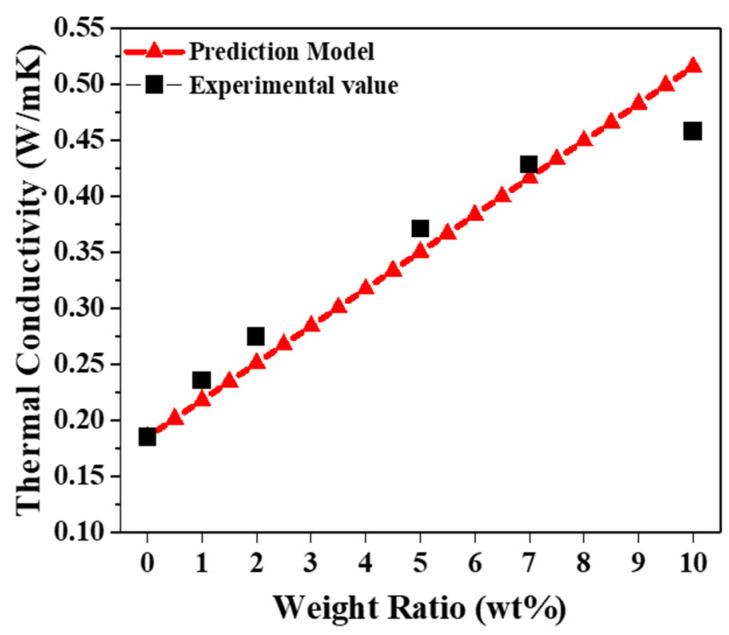
Comparison of the thermal conductivity from the prediction model (red triangles) and experimental values as a function of MWCNT wt% (black squares).

We substituted the thermal conductivity of pure PDMS obtained by experiment on this prediction value to show the thermal conductivity prediction value by weight ratio, which is expressed in a red triangle in Figure 6, to perform a comparison with the experimental value [42].

(3) $$\frac{k_{e}}{k_{m}}=1+\frac{nf/3}{\frac{k_{m}}{nk_{c}}+H\left(np\right)}\;,$$

where *k_e_* is the effective thermal conductivity of the composite; *k_m_* is the thermal conductivity of the polymer; *f* is the volume fraction of the filler; *k_c_* is the thermal conductivity of the filler; and *H*(*np*) is a factor that includes the influence of the aspect ratio. This is followed by Equation (4) [43]:

(4) $$H\left(np\right)=\frac{1}{{\left(np\right)}^{2}-1}\left[\frac{np}{\sqrt{{\left(np\right)}^{2}-1}}\ln\left(np+\sqrt{{\left(np\right)}^{2}-1}\right)-1\right],$$

where *p* is the aspect ratio of MWCNTs. This prediction model also considers the straightness of individual MWCNTs in the polymer matrix, which is expressed as *n* in Equation (3). MWCNTs exist in the form of a curve rather than a straight line inside the composite. In this case, the effective distance from one end of the curved MWCNTs to its other end is expressed as L_ce_, the actual length is L, and n is n = L_ce_/L [43]. The most appropriate value for our composite model was found to be n = 0.4. Based on this value, the comparison between the experimental and predicted values is shown in Figure 6. We confirmed that the predicted value of the model of composite thermal conductivity, Equation (3), and the experimental value matched well, indicating that the experiment was well performed. In addition, it could be seen that the comparison with the experimental value was performed by substituting the aspect ratio obtained in Figure 2d into Equation (4), which represents the effect of the aspect ratio on the thermal property of the composite. Experimentally, we observed that the higher the content of MWCNTs, the better the thermal conductivity. According to the results of the prediction model, the higher the content of MWCNTs in pure PDMS (0.1884 W/mK), with thermal conductivity at the level of insulation, the better the thermal conductivity linearly; the actual experimental data are also manifested according to this trend. This tendency is manifested in other previous studies; the thermal conductivity of this experiment (2 wt% = 0.2748 W/mK) was similar or a little better in the same mass ratio than the PDMS/MWCNT composite thermal conductivity of the previous experiments (2 wt% ≈ 0.2380 W/mK) [27].

## 4. Conclusions

In this study, we fabricated three different types of PDMS/MWCNT 1 wt% and 10 wt% composites with three different aspect ratios. Morphological analysis was performed to evaluate the length of the MWCNTs used and the degree of dispersion of the composite. The electrical and thermal conductivities of the composite were also measured. The experimental results showed that L-MWCNTs acted as excellent conductors, without contact resistance and phonon scattering. The L-MWCNTs required less filler content than S-MWCNTs to form a percolation network. S-MWCNTs required larger amounts of MWCNTs and contact points to form a percolation network, which led to increased interstitial contact resistance and poor physical properties. In addition, a comparative analysis was performed by introducing a predictive model suggested in previous studies to observe the effect of aspect ratio.

## Data Availability

Not applicable.
